# Chitosan Biguanidine/PVP Antibacterial Coatings for Perishable Fruits

**DOI:** 10.3390/polym14132704

**Published:** 2022-07-01

**Authors:** Xiangyu Jiao, Jiaxuan Xie, Mingda Hao, Yiping Li, Changtao Wang, Zhu Zhu, Yongqiang Wen

**Affiliations:** 1 Beijing Key Laboratory for Bioengineering and Sensing Technology, Daxing Research Institute, School of Chemistry and Biological Engineering, University of Science and Technology Beijing, Beijing 100083, China; s20200859@xs.ustb.edu.cn (J.X.); s20190855@xs.ustb.edu.cn (M.H.); liyiping1123@163.com (Y.L.); zhuzhu@ustb.edu.cn (Z.Z.); wyq_wen@ustb.edu.cn (Y.W.); 2 Key Laboratory of Cosmetic, China National Light Industry, Beijing Technology and Business University, Beijing 100048, China; wangct@th.btbu.edu.cn

**Keywords:** antimicrobial, chitosan derivative, coating, fruit

## Abstract

World hunger is on the rise, yet one-third of food is wasted. It is necessary to develop an effective food preservation method to reduce food waste. This article reports a composite film based on chitosan biguanidine hydrochloride(CBg) and poly (N-vinyl-2-pyrrolidone)(PVP) that can be used as a conformal coating for fresh produce. Due to the strong positive charge of CBg, the film has excellent antibacterial properties. Owing to the hydrogen bonds between CBg and PVP, the film has good flexibility and mechanical properties. In addition, the coating is washable, transparent, and can reduce the evaporation of water. The above characteristics mean the film has broad application prospects in the field of food preservation.

## 1. Introduction

As soils degrade, the planet warms, and the global population grows, world hunger is another issue that has been on the rise over the past years [1]. However, one-third of food is wasted [2], and a large part of this is due to the decay of food. Fresh agricultural products, such as fruits, only have a shelf life of a few days and are extremely perishable. Water loss, respiration, texture deterioration, and microbial growth are important factors affecting the life of these agricultural products after picking [3]. By controlling these factors, developing an effective solution to extend the preservation of agricultural products is essential to reducing food waste.

According to reports, the shelf life of perishable fruits can be improved by controlling some known factors, such as temperature, dehydration, and microbial growth [4,5]. Several preservation techniques are commercially used to extend the storage life of fruits, such as refrigeration and modified atmospheric packaging, which are the most common means of preserving fresh agricultural products [6]. However, these methods require energy consumption and special equipment, which makes the cost very high [7]. Other means, such as paraffin-based coatings or wax coatings, often affect the taste of fruits [8]. Therefore, it is very necessary to increase the shelf life of perishable foods without changing the biological and physical characteristics of them.

Recently, films made of proteins and polysaccharides have increasingly been used in the preservation of fruits because of their good biodegradability, edibility, and miscibility [9,10,11,12]. However, in terms of the many important requirements for fruit preservation, including preservation effect, material flexibility, washability, high transparency, and antibacterial properties, these materials have not shown comprehensive characteristics. Thus, it is necessary to develop a more functional film covering material.

Chitosan (CS) is a non-toxic and antibacterial natural polysaccharide. Its antibacterial activity is due to its positively charged amino groups that can interact with the negatively charged surface of bacteria [13,14,15,16]. However, the mechanical properties of CS film are very poor, which limits the application of CS. Cross-linking is an effective strategy to improve its mechanical strength. Therefore, chitosan is generally blended with other polymers, such as poly (vinyl alcohol) PVA, poly (N-vinyl-2-pyrrolidone) PVP, Poly (ethylene glycol) PEG, and so on, to expand its application range [17,18]. Nevertheless, CS has a low solubility of pH > 6.5 and only has strong antibacterial activity in acidic media, which limits the application of CS as a coating in the preservation of fruits [19].

Guanidine, a natural nitrogen-containing compound, has a strong positive charge in acidic, neutral, and alkaline solutions [20]. Thus, guanidine derivatives are considered to be excellent antibacterial materials [21]. Chitosan biguanidine hydrochloride (CBg), a guanidine derivative of CS, shows better solubility and antibacterial activity than CS. PVP, a widely used polyhydroxy polymer with good biocompatibility, good water solubility, and low cytotoxicity, is considered to have important application prospects in the production of coatings, plastics, detergents, cosmetics, and pharmaceuticals [22,23,24].

In this paper, we aim to prepare an antibacterial-coating-based CBg and overcome the limitations of chitosan-based antibacterial membranes. In order to improve the mechanical and water vapor barrier properties of the coating, PVP was chosen, as its amide groups can form hydrogen bonds with the amino groups (NH2) and the hydroxyl groups (OH) of CBg. The transparency, water solubility, water vapor barrier, mechanical, and antibacterial properties of the CBg/PVP coating were studied to evaluate its application in the preservation of strawberries. The main reasons strawberries were chosen are that it is a delicate fruit with excellent organoleptic properties and high nutritional value but an extremely short postharvest life. This film had excellent antibacterial properties due to the strong positive charge in CBg. Owing to the hydrogen bonds between CBg and PVP, the film had good mechanical and water vapor barrier properties. Moreover, the film also had good water solubility and transparency properties. In view of the above advantages, the film may have a good application prospect in the field of fruit and vegetable preservation.

## 2. Materials and Methods

### 2.1. Materials

Cyanoguanidine (99.99% purity) was purchased from Alfa Aesar Chemical Co., Ltd, MA, USA. CS (99.99% purity) was purchased from Hushi Chemical Co., Ltd, Shanghai, China. PVP (99.9% purity) was purchased from Aladdin Industrial Corporation, Beijing, China. Methanol and HCl were purchased from China National Medicines Corporation Ltd, Beijing, China Strawberries (Fragaria × ananassa Duch) without any post-treatment were obtained from a local orchard.

### 2.2. Synthesis of Chitosan Biguanidine Hydrochloride (CBg)

CBg was prepared by a previously reported procedure [25]. Briefly, 2 g chitosan (CS) powder was dissolved in 200 mL ultrapure water containing 1% hydrochloric acid, and then the mixture was heated in a water bath at 60 °C for 2 h. A 5 g amount of cyanoguanidine was dissolved in 50 mL ultrapure water and stirred evenly. Then, the cyanoguanidine solution was added to the chitosan solution dropwise at room temperature and reacted at 100 °C for 2 h. The reaction is shown in Scheme S1. After cooling to room temperature, the reaction mixture was precipitated in excess methanol, then filtered and dialyzed with ultrapure water for 3 days to remove the unreacted reagent, and then freeze-dried for 4 days to prepare the white chitosan biguanidine hydrochloride (CBg) powder.

### 2.3. Preparation of CBg/PVP Film

A 3 g amount of PVP powder was dissolved in 100 mL ultrapure water and stirred for 2 h at room temperature to obtain 3% PVP solution. Then, different proportions of CBg were added to 3% PVP solution and stirred for 1 h. After that, the mixed solution (15 mL) was coated on the PTFE mold (diameter 7 cm) and placed in a constant temperature oven at 60 °C for 24 h to obtain CBG/PVP films with different mass concentrations.

### 2.4. Spectral Characterization of CBg and CBg/PVP Films

The infrared spectra of CS, CBg, and CBg/PVP films with different mass concentrations were measured by FTIR in the range of 3600–800 cm^−1^ The transmission spectrum of the CBg/PVP film was analyzed with an ultraviolet spectrophotometer in the range of 300~1100 cm^−1^.

### 2.5. Mechanical Properties of CBg/PVP Membrane

The tensile properties of CBg/PVP films were measured by ESM 303 (Mark-10 Corporation, NY, USA). First, the film was cut into 10 mm × 30 mm strips. The cut film was clamped on the tensile testing machine. The effective tensile length was 10 mm, and the tensile rate was 20 mm/min. The film thickness was controlled at 0.1 ± 01 mm, which was measured by a screw micrometer. Each sample was tested 5 times.

### 2.6. Water Vapor Permeability

WVP was measured according to ASTM E96-92. The film was cut into a circle with a diameter of 7 cm. The film was placed over a 7 cm (diameter) permeability cup with 30 mL of ultrapure water, and the liquid level was kept 2 cm away from the film. Then, the dish was placed in a constant temperature and humidity box at 23 °C and 50% humidity, and the change in dish mass was recorded every 24 h. The WVP calculation formula is as follows:

$$\mathrm{WVP}=\frac{24M}{\mathrm{At}}$$

where M (g) is the weight of evaporated water, t (h) is the evaporation time, and A (m^2^) is the area of the test film.

### 2.7. Antibacterial Experiment of CBg/PVP Film

The antimicrobial properties of CBg/PVP films were tested using previously published literature methods [26,27]. In short, suitable strains of *Escherichia coli* (*E. coli*) and *Staphylococcus aureus* (*S. aureus*) were selected and co-cultured with 20 mL liquid medium at 37 °C overnight to obtain a microbial suspension (~1010 CFU/mL). After diluting with phosphate-buffered saline (PBS) solution, the microbial suspension (~106 CFU/mL) was mixed with CBg/PVP film (2 cm^2^) with different mass concentrations for 24 h at 37 °C. Afterward, every 100 μL of the mixed suspension was coated on the solid medium and cultured in a constant temperature biochemical cabinet at 37 °C for 24 h. At last, the colonies were counted with an automatic colony counter.

### 2.8. Solubility

To investigate the solubility performance of the CBg/PVP film, dry CBg/PVP films were immersed in ultrapure water in a conical flask and gently stirred at room temptation for 0.5 h. Then, the films were transferred to a dish and dried in a vacuum oven for 3 h (100 °C). Finally, the weight of the films was measured three times. Sol% was calculated as follows:

$$\mathrm{Sol}\;\left(\%\right)=\frac{M_{0}-M_{1}}{M_{0}}$$

where *M*_0_ is the weight of the film before being immersed in water and after drying in vacuum, and *M*_1_ is the weight of the film after immersion in water and drying in vacuum.

### 2.9. Application to Strawberries

Strawberries of similar quality and without any defects were soaked completely in the composite solution by holding the stem. Then, the strawberries were hung on a shelf and dried at room temperature for 2 h.

The mass loss of strawberries was carried out in an indoor environment at about 22 °C and 35% RH. Each set of samples used eight strawberries. The weight loss of each strawberry was measured at 24 h intervals. The average weight of each strawberry was taken after three measurements. The mass loss percentage was calculated as follows:

$$\mathrm{Mass}\;\mathrm{loss}=\frac{m-m_{0}}{m_{0}}$$

where *m* is the weight at subsequent days after storage, and *m*_0_ is the initially weight after coating.

The firmness of fruits was assessed by a fruit hardness tester (GY-4, HANDPI, Wenzhou, China).

## 3. Results

### 3.1. Fourier Transform Infrared (FTIR) Spectroscopy

**Figure 1:**
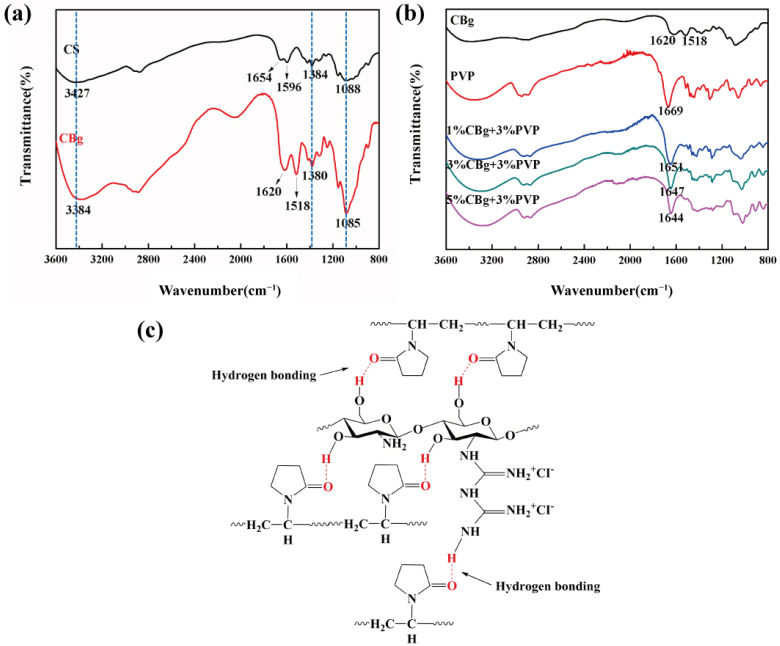
(**a**) FTIR spectra of CS and its derivative CBg. (**b**) FTIR spectra of CBg, PVP, and CBg/PVP films with different mass concentrations. (**c**) Schematic diagram of the interaction between CBg and PVP.

The FTIR spectrum of CS and its derivative CBg is shown in Figure 1a. There is a wide absorption band at 3427 cm^−1^ due to the stretching vibration of -OH and -NH_2_ on the CS backbone chain. At 1654 cm^−1^ and 1596 cm^−1^ are the characteristic absorptions of amide I and II, respectively [28]. The C-N stretching vibration of CS aromatic carbon is located at 1384 cm^−1^. The absorption peak at 1080 cm^−1^ is attributed to the C-O stretching vibration of CS. Compared to CS, the absorption peak of CBg at 3384 cm^−1^ was enhanced, indicating that the N-H content of the derivative was increased [29]. However, the absorption peak at 1620 cm^−1^ is attributed to the -C=N- stretching vibration in CBg, and the increase in the peak indicates the increase in the secondary amine groups in the product [25]. The absorption peak at 1518 cm^−1^ is attributed to the -NH_2_^+^ bending vibration in CBg [21]. The absorption peak at 1085 cm^−1^ is enhanced, indicating the C-O stretching vibration and the alkyl carbon C-N stretching vibration in CBg. These new absorption bands and the peak enhancement of the original absorption bands all confirmed the successful synthesis of CBg.

FTIR is often used to determine the interaction between CS and counterpart polymers. Both the amino groups (NH_2_) and the hydroxyl groups (OH) can form hydrogen bonds with the amide groups of PVP [30]. The FTIR spectra of the CBg, PVP, and CBg/PVP films with different mass concentrations are shown in Figure 1b. As shown in the FTIR spectrum of PVP, its characteristic peak is located at 1669 cm^−1^, which is attributed to the amide carbonyl group. With the increase in CBg content, the interaction between PVP and CBg was enhanced, and the characteristic absorption peak gradually moved to a low frequency. The characteristic absorption peak of the 1% CBg + 3% PVP film (1CBg/PVP) moved to 1651 cm^−1^. The characteristic peak of the 3% CBg + 3% PVP (3CBg/PVP) film moved to 1647 cm^−1^. The characteristic peak of the 5% CBg + 3% PVP film (5CBg/PVP) moved to 1644 cm^−1^. The peak shift indicates the incidence of interaction between CBg and PVP. This interaction is attributed to the hydrogen bonds formed between the proton donor hydroxyl and the amino groups of CBg and the proton acceptor C=O of PVP [31], as shown in Figure 1c.

### 3.2. The Transmission Spectrum of the CBg/PVP Film

**Figure 2:**
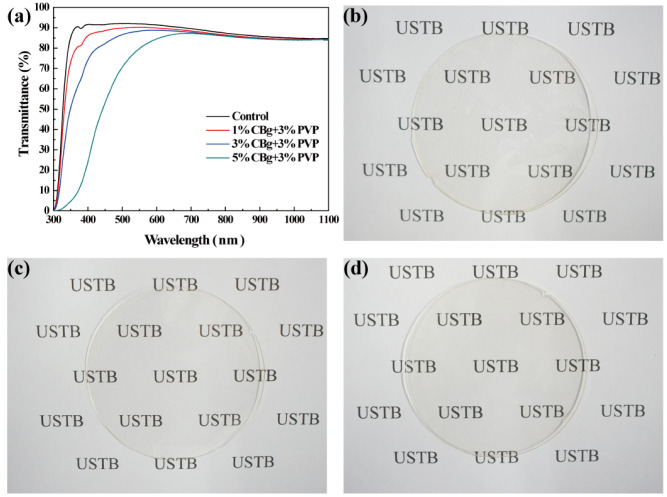
(**a**) UV-vis transmission spectrum of CBg/PVP films with different mass concentrations. Photographs of (**b**) 1CBg/PVP, (**c**) 3CBg/PVP, and (**d**) 5CBg/PVP films.

Fruit coatings need to have good light transmittance so as to not affect the appearance of food. The CBg/PVP films were coated on glass to test the UV-vis transmission spectrum. As shown in Figure 2a, compared to glass (control), the transmittance of glass coated with different CBg/PVP films in the visible range (380 to 750 nm) is reduced. The decrease in the transmittance of 1CBg/PVP and 3CBg/PVP in the visible light range was acceptable. However, the transmittance of 5CBg/PVP in the visible range decreased more. Furthermore, the photographic images of the CBg/PVP films confirmed that the addition of a small amount of CBg had little effect on the transparency of the film (Figure 2b–d).

### 3.3. Film Thickness

Film thickness is an important parameter of fruit coatings that affects the WVP and mechanical properties of the films. The thicknesses of the films are shown in Table S1. In general, the thicknesses of all of the films are almost the same, around 80 μm.

### 3.4. Mechanical Properties

**Figure 3:**
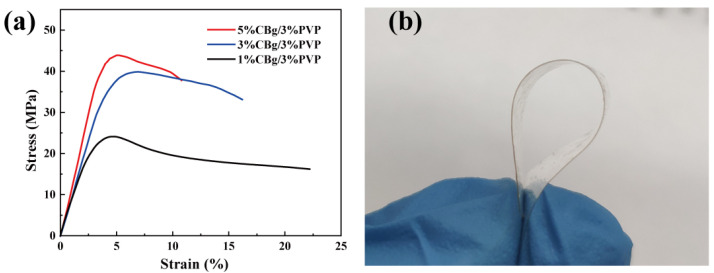
(**a**) Tensile testing of CBg/PVP films with different mass concentrations. (**b**) Photograph of 3% CBg/3% PVP film.

Fruit coatings should have good mechanical properties to avoid cracking during transportation and storage. Figure 3a shows the average stress–strain curve. Young’s modulus (YM), the extracted tensile strength (TS), and the elongation at break (EB) of the films are listed in Table 1. YM was calculated in the strain range of 0.4% to 0.32%, R^2^ > 0.98. With the increase in CBg content, the YM of the film increased from 6.5 MPa (1CBg/PVP) to 10.16 MPa (5CBg/PVP), which may be due to the hydrogen bonding between CBg and PVP that increased the cross-linking of the polymer chains. In addition, as the film strength increased, TS also increased when the CBg content increased. EB was decreased from 23.28 (1CBg/PVP) to 13.25 (5CBg/PVP), which may be due to polymer chains that were partially immobilized because of the cross-linking reaction. The above results indicate that the film has excellent mechanical properties. In addition, the film is extremely flexible, as it can be bent and folded repeatedly without breaking, as shown in Figure 3b.

### 3.5. Water Vapor Permeability

The ideal preservative coating needs to have a lower WVP value to delay the loss of water from food. As shown in Table 1, the WVP values of the CBg/PVP films were 301.17 ± 11.17 (1CBg/PVP), 154.21 ± 15.66 (3CBg/PVP), and 138.64 ± 15.12 (5CBg/PVP), and the higher CBg content, the lower the water vapor transmission rate. This may be due to the cross-linking between PVP and CBg forming an interconnected network.

### 3.6. Solubility

**Figure 4:**
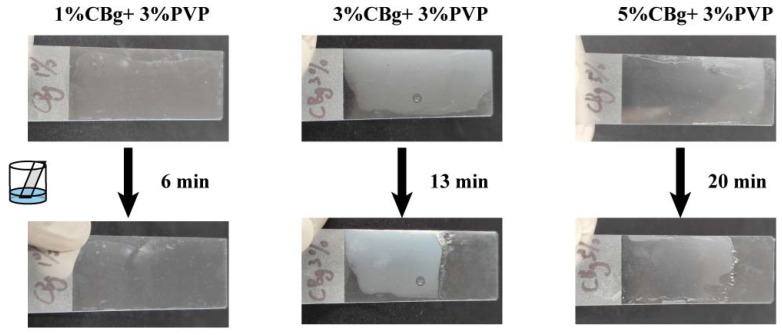
The times for CBg/PVP films with different mass concentrations to separate them from the glass in stirred water.

Consumers may prefer the taste of uncoated fruit, so we tested the solubility percentage (Sol%) of the films over 0.5 h. The results are shown in Table S2. The Sol % of the CBg/PVP films decreased with the increase in CBg content. This decrease in solubility may be due to the cross-linking of hydrogen bonds forming a network matrix. We also coated the films on glass and immersed part of the glass in vigorously stirred water to test how long it took the soaked part of the film to peel off from the glass surface. As shown in Figure 4, after being immersed in stirred water for 6 min, the 1CBg/PVP film peeled off from the glass, while the 5CBg/PVP film needed to be soaked for a longer time (20 min) to peel off from the glass. This is consistent with the solubility experiment above. In addition, gentle scrubbing can make it easier for the 3CBg/PVP film to separate from the glass. As shown in Video S1, after soaking for several tens of seconds, the film can be peeled off from the glass after gentle rubbing. The experimental results show that compared to the non-washable wax coating currently used, the CBg/PVP coating can be easily removed through washing and gentle rubbing.

### 3.7. Antibacterial Activity

**Figure 5:**
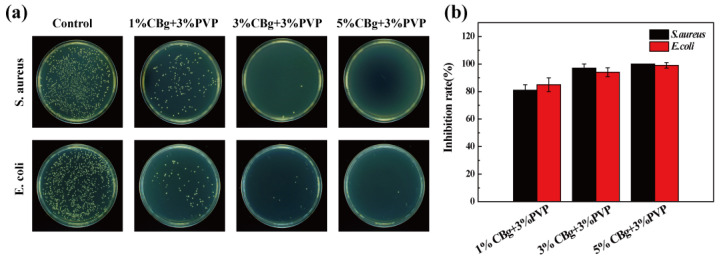
(**a**) Antibacterial effect of different CBg/PVP films on *S. aureus* and *E. coli*. (**b**) Inhibition rate of different CBg/PVP films.

Through the co-cultivation method, the antibacterial effects of CBg/PVP films with different mass concentrations on Gram-positive *S. aureus* and Gram-negative *E. coli* were studied. As shown in Figure 5a and Figure S1, the blank group was full of bacteria, while the treatment groups showed a certain antibacterial effect. With the increase in CBg content, the antibacterial property of the film increased. This may be due to the increased density of the charges in the film, which can interact strongly with the bacterial wall to change the permeability of the bacteria and cause it to lyse and die [28]. The 5% CBg + 3% PVP film showed the best antibacterial effect, as shown in Figure 5b.

### 3.8. Application to Strawberries

**Figure 6:**
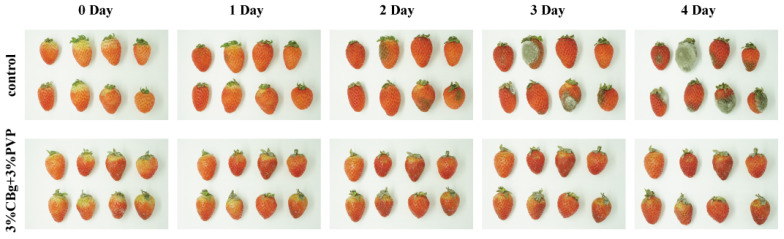
Photographic images of strawberries coated/uncoated with 3CB/PVP films.

Based on the above experimental results and cost considerations, the 3CBg/PVP film was selected for the strawberry preservation experiment. The strawberries were coated using the “dipping method”, and the freshness preservation effect of the film was further evaluated. The surfaces of the coated strawberries were inspected every 24 h to observe their spoilage, and the results were compared with the uncoated strawberries. As shown in Figure 6, the surface of the uncoated strawberries showed mold growth after 2 days. The strawberries coated with the 3CBg/PVP film showed no mold growth, even at 4 days, while the uncoated strawberries had been covered by a large area of mold. These results confirmed the ability of the 3CBg/PVP film to inhibit bacterial growth.

Figure S2 shows the mass loss in 3CBg/PVP-coated strawberries and uncoated strawberries. The mass loss in uncoated strawberries increased significantly with the extension of the storage time. This mass loss is due to the evaporation of water from the strawberries into the environment. The mass loss in coated strawberries was significantly lower than that of uncoated strawberries. This is because the existence of the membrane barrier effectively reduces the evaporation of water.

Figure S3 shows the decrease in the firmness of all of the strawberries after storage for 4 days. Particularly, the firmness of the strawberries in the control group reduced faster than those coated with the 3CBg/PVP film, which indicated that the films prolonged the softening rate of the strawberries. The loss in hardness may be due to the degradation of the cell wall caused by the dehydration process [32]. The results showed that the limited water vapor permeability of the CBg/PVP coating reduced the transfer of water vapor from the fruit, delaying the degradation process.

## 4. Conclusions

Food waste is a major obstacle to eliminating global hunger, and extending the shelf life of food through preservation methods is a direct way to solve this problem. Antibacterial films based on CBg and PVP were prepared in this paper. The films showed excellent comprehensive characteristics, including good flexibility, mechanical properties, transparency, water vapor barrier properties, was hability, and excellent antibacterial properties. The verification experiment of the strawberry model shows that the coating can greatly increase the shelf-life time of strawberries. We believe that the fruit coatings prepared in this work have certain application prospects for solving the global food waste problem.

## Figures and Tables

**Table 1:** Mechanical properties and WVP of CBg/PVP films with different mass concentrations.

	YM (MPa)	TS (MPa)	EB (%)	WVP (g/m^2^/day)
1CBg/PVP	6.50 ± 0.45 ^a^	19.85 ± 3.50 ^a^	23.28 ± 1.41 ^a^	301.17 ± 11.17 ^a^
3CBg/PVP	8.74 ± 0.55 ^b^	33.32 ± 2.84 ^b^	17.87 ± 0.61 ^b^	154.21 ± 15.66 ^b^
5CBg/PVP	10.16 ± 0.16 ^c^	41.43 ± 2.33 ^c^	13.25 ± 1.02 ^c^	138.64 ± 15.12 ^b^

Values with different letters (a, b and c) are significantly different (*p* < 0.05) while those with similar letters (a, b and c) are non-significantly different (*p* > 0.05).

## Data Availability

The data supporting the findings of this study are available from the corresponding author upon reasonable request.

## Supplementary Materials

The following supporting information can be downloaded at: https://www.mdpi.com/article/10.3390/polym14132704/s1, Scheme S1: Schematic diagram of the reaction between chitosan and cyanoguanidine. Figure S1: Antibacterial effect of different CBg/PVP film on *S. aureus* and *E.* coli. Figure S2: Storage mass loss of uncoated and coated strawberries. Figure S3: Storage mass loss of uncoated and coated strawberries. Table S1: Thickness of different CBg/PVP films. Table S2: Sol % of CBg/PVP films in 0.5 h; Video S1.
