# Supplementary material for: Chitosan Biguanidine/PVP Antibacterial Coatings for Perishable Fruits
Source: Polymers (Basel). 2022 Jul 1;14(13):2704. doi: 10.3390/polym14132704 (PMC9269116; doi:10.3390/polym14132704)
Supplement: Supplementary file 1 [file polymers-14-02704-s001.zip › Supplementary Files/supplementary.pdf]

Chitosan biguanidine/PVP antibacterial coatings for perishable fruits

Xiangyu Jiao[a,*], Jiaxuan Xie[a], Mingda Hao[a], Yiping Li[a], Changtao Wang[b], Zhu Zhu[a], and Yongqiang Wen[a]

a Beijing Key Laboratory for Bioengineering and Sensing Technology, Daxing Research Institute, School of Chemistry and Biological Engineering, University of Science and Technology Beijing, Beijing 100083

b Key Laboratory of Cosmetic, China National Light Industry, Beijing Technology and Business University, Beijing 100048

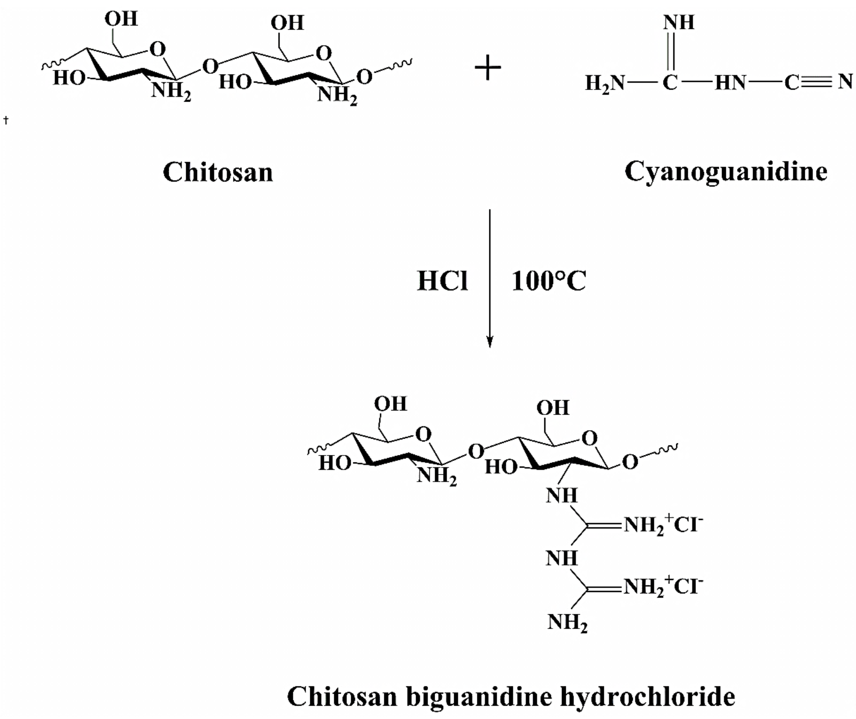

Scheme S1. Schematic diagram of the reaction between chitosan and cyanoguanidine

	1% CBg/ 3% PVP	3% CBg/ 3% PVP	5% CBg/ 3% PVP	*p*
Thickness (μm)	80±7	87±10	78±10	0.27

At the 0.05 level, the population means are not significantly different.

Table S1. Thickness of different CBg/PVP films

	1% CBg/ 3% PVP	3% CBg/ 3% PVP	5% CBg/ 3% PVP
Sol %	46.3	44.4	40.9

Table S2. Sol % of CBg/PVP films in 0.5 h

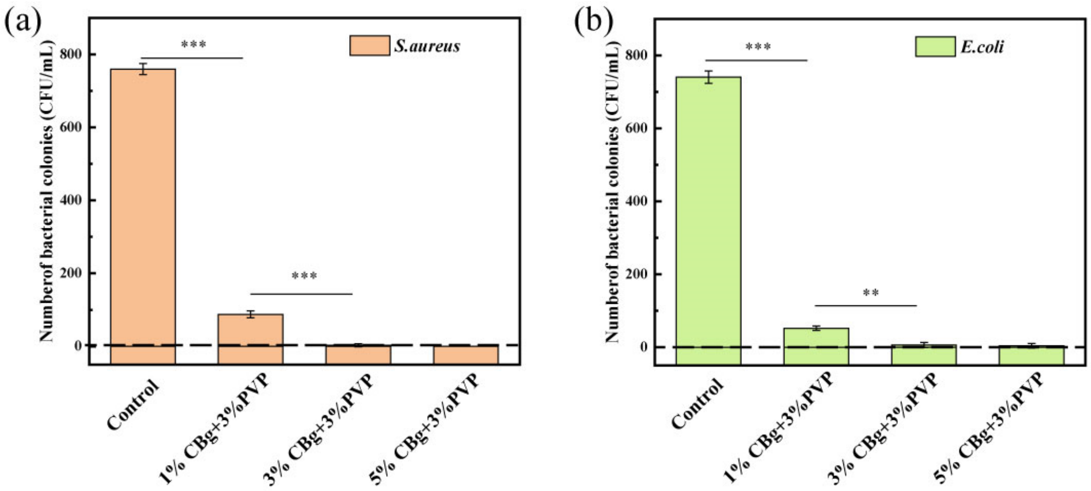

Figure S1 Antibacterial effect of different CBg/PVP film on S. aureus and E. coli. * $p < 0.05$, ** $p < 0.01$ and *** $p < 0.001$.

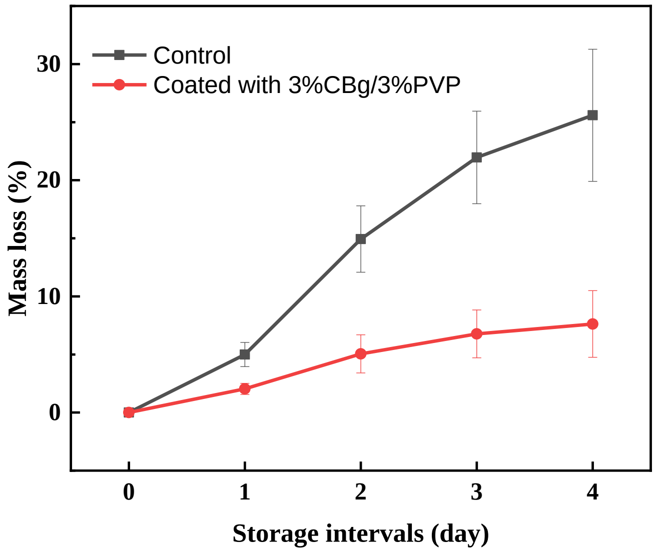

Figure S2 Storage mass loss of uncoated and coated strawberries.

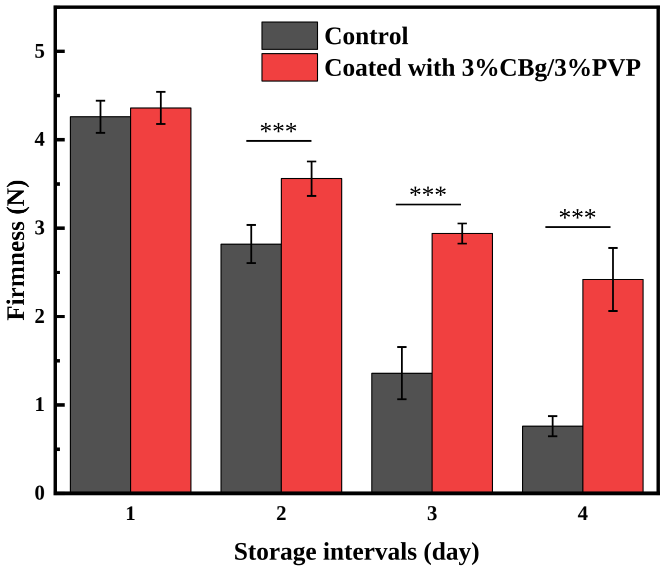

Figure S3 Storage mass loss of uncoated and coated strawberries. * $p < 0.05$, ** $p < 0.01$ and *** $p < 0.001$.
